# The diagnostic value and safety of modified needle aspiration biopsy for superficial lymphadenectasis

**DOI:** 10.3389/fsurg.2022.968706

**Published:** 2022-10-25

**Authors:** Shaopeng Hua, Xiaofang Hu, Xinguo Zhao, Jia Mao

**Affiliations:** ^1^ Department of Tuberculosis, Wuxi Fifth People's Hospital, Wuxi, China; ^2^ Department of Eemergency, The Affiliated Wuxi No. 2 People's Hospital of Nanjing Medical University, Wuxi, China

**Keywords:** modified needle aspiration biopsy, core needle biopsy, lymph node enlargement, diagnostic value, security

## Abstract

**Objective:**

To explore the safety and diagnostic value of modified needle aspiration biopsy for superficial enlarged lymph nodes.

**Methods:**

A total of 88 patients with unknown diagnosis of superficial lymphadenopathy in our hospital (Wuxi Fifth People's Hospital) from January 2018 to July 2021 were recruited and then divided into two groups using the simple random grouping method. The study group included 48 patients who were punctured *via* modified needle aspiration biopsy, while the control group included 40 patients who were punctured *via* core needle biopsy (CNB) and had a better clinical evaluation. A BARD® automatic biopsy gun with a 18 G needle was used to puncture any enlarged lymph nodes, and specimens were obtained for pathological examination. The diagnostic positive rate of the two puncture methods was then compared and the complications involved in the two methods were observed.

**Results:**

The positive rate of definite pathological diagnosis was 79.2% (38/48) in the study group and 82.5% (33/40) in the control group. Both groups had similar diagnostic positive rates regarding tuberculosis and metastatic tumours in the lymph nodes (*P* > 0.05). Only slight bleeding was observed during the operations using the two puncture methods, with the bleeding rate of the study group 20.8% and that of the control group 15.0%, and the difference was not statistically significant (*P* > 0.05). No other complications were observed across both groups. Among the 48 patients with enlarged lymph nodes in the study group, there was no difference in the positive rate of diagnosis between enlarged lymph nodes larger than 20 mm and those smaller than 20 mm in the pathological, bacterial culture and cytology examinations (*P* > 0.05). The sensitivity, specificity, positive predictive values and negative prediction values of the improved lymph node lesions were 83.3%, 100%, 100% and 90.9%, respectively.

**Conclusion:**

The diagnostic efficacy and safety of modified needle aspiration biopsy in superficial swollen lymph nodes are equivalent to those of CNB, but the former is a more economical, simple and practical method for clinical settings and one that could be popularised in primary hospitals.

## Introduction

Lymphadenectasis is a clinically common lesion and may occur due to a variety of causes, including infection, inflammation, tumour occurrence or simply non-specific reactive hyperplasia (1). Currently, it remains a serious physical and mental health risk in many developing countries (2). Accurate positioning and qualitative identification of abnormal lymph nodes are crucial for the attendant treatment option selection, the later efficacy evaluation and the prognostic follow-up (3).

The final diagnosis of lymphadenectasis requires a pathological diagnosis (4). At present, lymph node pathological tissue is mainly obtained through surgical lymph node resection biopsy, fine needle aspiration cytology (FNAC), core needle biopsy (CNB) or other methods (5). Surgical lymphadenectomy biopsy has high sensitivity and specificity, but its application in disease evaluation is controversial due to its long operation time and high cost, as well as the possibility of major traumatic damage and more sequelae (6, 7). While FNAC has long been used as a diagnostic tool due to its high safety (8), the method allows for obtaining few tissues, and certain specimens can only undergo simple cytology, which cannot meet the requirements of a large sample size for a clear pathological diagnosis of the disease (9, 10). Meanwhile, CNB has the advantages of less trauma, fewer complications and higher diagnostic accuracy than FNAC (11), while the method allows for obtaining sufficient pathological tissue for a histopathological diagnosis that presents more clinical recommendations (12–14). However, the puncture needle used is expensive, and this can easily increase the medical costs.

Transbronchial needle aspiration (TBNA) is a new technique that involves inserting a special puncture needle with a flexible catheter into the airway through the bronchoscopy biopsy channel. The puncture needle penetrates the airway wall, attracts any tracheal and bronchial lesions outside the lumen, such as nodules and enlarged lymph nodes, and obtains relevant specimens for cytology and histopathological examination (15). Inspired by the TBNA technique, we modified the traditional FNAC method by increasing the negative needle suction pressure to 20 ml and the thickness of the needle to 18 G. The effect of our modified needle aspiration biopsy method in the diagnosis of superficial lymph nodes was compared to that of CNB in view of assessing the diagnostic value and safety of the new method in terms of superficial lymphadenectasis. Overall, the modified needle aspiration biopsy method demonstrated a similarly high diagnostic rate as CNB.

## Data and methods

### General information

This study selected 88 patients with lymphadenectasis who were admitted to Wuxi Fifth People's Hospital from January 2018 to July 2021. This included 52 males and 36 females aged 11–86 years, with a mean age of 48 ± 20 years. Among the 88 patients, there were 56 cases of neck lymphadenectasis, 20 cases of supraclavicular lymphadenectasis, four cases of axillary lymphadenectasis and eight cases of submaxillary lymph nodes. The recruited patients were randomly divided into two groups, with 48 patients in the study group (modified needle aspiration biopsy group) and 40 in the control group (CNB group). The study was approved by the Ethics Committee of Wuxi Fifth People's Hospital (Institutional Review Board number: 2017-053-1; Institutional Review Board Date: 2017-12-1), and all the patients signed an informed consent prior to the surgery.

### Inclusion and exclusion criteria

The inclusion criteria were as follows: (i) patients who required a pathological examination of lymph node biopsy due to unclear diagnosis; (ii) patients with enlarged superficial lymph nodes that could be detected *via* ultrasound examination; (iii) patients with enlarged superficial lymph nodes that were palpable and (iv) patients with enlarged superficial lymph nodes with no symptoms of rupture, bleeding or pus flow.

The exclusion criteria were as follows: (i) patients with mental illness; (ii) patients with systemic infectious diseases; (iii) patients with severe cardiopulmonary disease and coagulation dysfunction, with a recent history of myocardial infarction and cerebrovascular accident; (iv) patients with an intolerance to local anaesthesia or who were allergic to narcotic tics and (v) patients who were unwilling to sign the informed consent form.

### Methods

Prior to the surgery, the patients underwent blood routine, coagulation function and colour Doppler ultrasound (GE E9 colour Doppler ultrasound diagnostic instrument, General Company) examinations for detection of enlarged superficial lymph nodes, with the puncture point and path of the lymph nodes initially determined. Prior to the puncture procedure, a conventional puncture package was prepared, which included a sterile gauze, sterile gloves, a 5-ml syringe, a 20-ml syringe, a BARD® automatic biopsy gun with an 18 G puncture biopsy needle, 2% lidocaine, a 10% neutral formalin solution, glass slides and a sterile container. The study group underwent modified needle aspiration biopsy, while the control group underwent CNB.

#### Modified needle aspiration biopsy procedure

**Figure 1:**
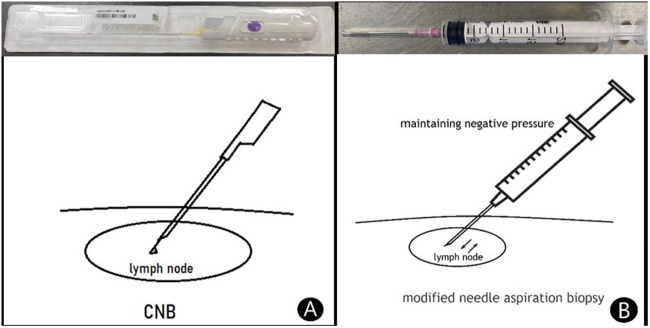
The visuals of both methods. (**A**) Core needle biopsy (CNB). Use Bard automatic biopsy gun with 18G biopsy needle to directly puncture enlarged lymph nodes for biopsy. (**B**) Modified needle aspiration biopsy. Use a 20 ml syringe with an 18G needle to puncture into the enlarged lymph node, and then maintain 20 ml negative pressure for repeated aspiration 15–20 times for needle biopsy.

The puncture lymph node was fixed with the left hand following local anaesthesia. The 18-G needle with a 20-ml syringe was then connected using the right hand and was inserted using the proposed puncture point and puncture path. Once the needle had penetrated the lymph node, the assistant drew 20 ml of negative pressure from the syringe and maintained it. According to the preoperative depth of the puncture, a 20-ml negative pressure was repeatedly aspirated 15–20 times, and the operators observed whether there was blood withdrawal in the negative pressure syringe. Following the needle aspiration biopsy, the assistant continued to maintain the negative pressure until the operator completely withdrew the puncture needle (Figure 1B). Specimens were then added to the 10% neutral formalin solution, to the glass slides or into the sterile container. Needle aspiration biopsies were repeated 3–5 times and samples were submitted for pathological tissue, cytology and bacteriological culture examinations.

#### Core needle biopsy

Following local anaesthesia, the biopsy gun was used to penetrate the enlarged lymph node, and a safe distance was measured. The biopsy gun was then set to cut and puncture 2–3 times (Figure 1A). The puncture tissue was subsequently fixed with the 10% neutral formalin solution and submitted for pathological tissue examination.

### Evaluation indicators

#### Definitive pathological diagnosis rate

For the enlarged lymph node biopsy, rapid paraffin sections or rapid frozen sections were prepared and the tissue sections were observed for histological diagnosis. The biopsy samples were examined *via* bacterial culture and cell smear procedures for microbiology and aspiration cytology diagnosis. A definitive diagnosis rate was calculated based on the final diagnosis and the pathological diagnosis of lymphadenectomy.

#### Evaluation indicators of diagnostic test value

To determine the authenticity, reliability and practicability of reaction modified needle aspiration biopsy for the diagnosis of lymphadenectasis, the sensitivity, specificity, positive predictive value and negative predictive value for identifying benign or malignant superficial lymphadenectasis were analysed, with the pathological diagnostic results of the lymph node resection adopted as the diagnostic criteria.

#### Complications

The only complication was bleeding at the puncture site, with no other complications occurring during the procedures.

### Statistical analysis

The statistical analysis was performed using SPSS17.0 software. The measurement data were assessed for normal distribution using the Kolmogorov–Smirnov test, with those meeting the normal distribution expressed as mean ± standard deviation and an independent sample *t*-test used for inter-group comparisons. The data not following normal distribution were expressed in terms of median and interquartile spacing, with inter-group comparisons performed using the Mann–Whitney *U*-test. Finally, the categorical variables were expressed in terms of number (%) and were analysed using Fisher's exact test. A *P*-value of <0.05 indicated a statistically significant difference.

## Results

### Baseline characteristics

The baseline data of the patients included in this study are shown in Table 1. A total of 88 cases were included, with 48 patients in the research group, including 28 males and 20 females with an average age of 50 ± 20 years. In this group, the diameter of the lymph nodes was 26.5 ± 7.8 mm. Of the 40 patients in the control group, 24 were male and 16 were female, with an average age of 46 ± 19 years. In this group, the lymph node diameter was 26.7 ± 10.6 mm. Various demographic characteristics, including body mass index, history of underlying disease, pathological diagnosis and family history of malignancy were also recorded for both groups, with the details shown in Table 1. The results of the statistical analysis revealed that the differences in the baseline data were not statistically significant (*P* > 0.05), indicating that the two groups were comparable.

**Table 1:** General data of patients included in the study.

General information	Control group	Research group	*P* value
Number of cases included (*n*)	40	48	
Age (year)	46 ± 19	50 ± 20	0.228
Gender			0.874
Male	24 (60.0%)	28 (58.3%)	/
Female	16 (40.0%)	20 (41.7%)	/
Body mass index (BMI)	21.4 ± 1.5	21.7 ± 1.8	0.769
History of underlying disease	14 (35%)	19 (39.6%)	0.196
Puncture diameter (mm)	26.7 ± 10.6	26.5 ± 7.8	0.854
Pathological diagnosis			1.389
Benign	20 (50%)	30 (62.5%)	
Malignant	20 (50%)	18 (37.5%)	
Family history of malignant tumors	4 (10%)	6 (12.5%)	0.135

### Comparison of pathological tissues

**Figure 2:**
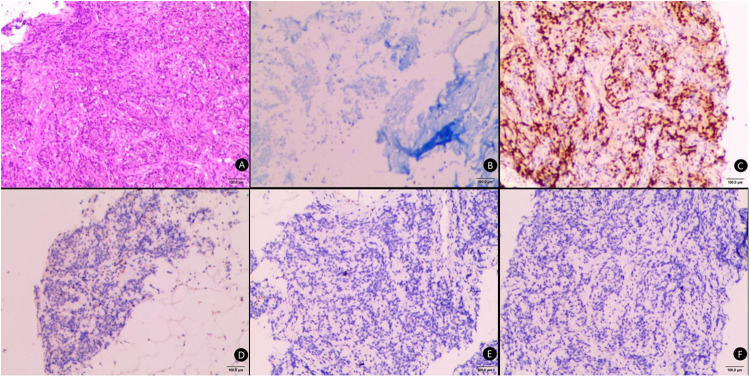
Pathological tissue examination of the modified needle aspiration biopsy specimens. (**A**) Acid-fast staining of the puncture tissue, bar = 100μm; (**B**) CgA staining of the puncture tissue, bar = 100μm; (**C**) P40 staining of the puncture tissue, bar = 100μm; (**D**) HE staining of the puncture tissue, bar = 100μm; (**E**) CK7 staining of the puncture tissue, bar = 100μm; (**F**) TTF1 staining of the puncture tissue, bar = 100μm.

**Figure 3:**
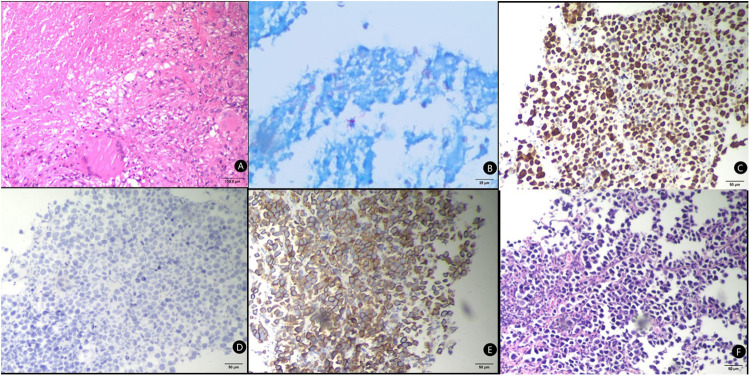
Pathological tissue examination of core needle biopsy specimens. (**A**) HE Staining of the puncture tissue, bar = 100μm; (**B**) acid-fast staining of the puncture tissue, bar = 25μm; (**C**) Ki67 Staining of the puncture tissue, bar = 50μm; (**D**) CgA Staining of the puncture tissue, bar = 50μm; (**E**) CK7 Staining of the puncture tissue, bar = 50μm; (**F**) TTF-1 Staining of the puncture tissue, bar = 50μm.

The puncture specimens of the study group were mainly broken tissues, with few complete tissues. Pus or pus and blood secretions could be extracted from some of the infectious enlarged lymph nodes. The number of specimens met the requirements for pathological tissue, cytological and bacteriological culture examinations, while an immunohistochemical evaluation of the tumour tissues could also be performed (Figure 2). The puncture specimens in the control group were mainly intact tissue strips and all the specimens also met the requirements of pathological examination and immunohistochemical evaluation (Figure 3).

### Comparison of diagnostic rates

The diagnostic results for the two groups are presented in Table 2. In the study group, based on the pathological diagnosis of lymph node resection, there were 23 cases of tuberculosis, 16 cases of metastatic tumours, six cases of reactive hyperplasia, one case of necrotising lymphadenitis and two cases of lymphoma. The modified needle aspiration biopsy confirmed 38 cases (38/48), with a diagnostic rate of 79.2%, including 18 cases of tuberculosis (18/23, 78.2%), 15 cases of metastatic tumours (15/16, 93.8%), four cases of reactive hyperplasia (4/6, 66.7%) and one case of necrotising lymphadenitis (1/1, 100%), while there were zero cases of no lymphoma (0/2, 0%). Meanwhile, in the control group, again based on the pathological diagnosis of lymph node resection, there were 19 cases of tuberculosis, 18 cases of metastatic tumours, one case of reactive hyperplasia and two cases of lymphoma. A total of 33 cases (33/40) were identified *via* CNB, with a diagnostic rate of 82.5%, including 15 cases of tuberculosis (15/19, 78.9%), 17 cases of metastatic tumour (17/18, 94.4%), one case of reactive hyperplasia (1/1, 100%) and zero cases of no lymphoma (0/2, 0%).

**Table 2:** Comparison of pathological diagnosis results between the two groups.

		Tuberculosis	Metastatic tumor	Reactive hyperplasia	Necrotic lymphadenitis	Lymphoma	Total
Research group	Number of diagnoses/total cases (*n*)	18/23	15/16	4/6	1/1	0/2	38/48
	Diagnostic rate	78.2%	93.8%	66.7%	100%	0%	79.2%
Control group	Number of diagnoses/total cases (*n*)	15/19	17/18	1/1	0/0	0/2	33/40
	Diagnostic rate	78.9%	94.4%	100%	/	0%	82.5%
*P* value		1.000	1.000	1.000	/	/	0.693

The positive rate of definitive pathological diagnosis was lower in the study group than in the control group, but the difference was not statistically significant (*P* > 0.05). The positive rates of lymph node tuberculosis and metastatic tumours were similar between the two groups, with the difference not statistically significant for both rates (*P* > 0.05). It was therefore concluded that modified needle aspiration biopsy can achieve the same performance as CNB in terms of diagnostic rate. However, both methods did not correctly diagnose lymphoma.

### Comparison of diagnosis effect of aspiration cytology and microbiological examinations

Cytology and microbiological diagnoses were performed for the two groups of lymph node tissues, with the results shown in Table 3. In the study group, a bacterial culture procedure was performed on the specimens pertaining to the modified needle aspiration biopsy procedure. Here, there were 10 positive cases of acid-resistant bacilli (10/23, 43.5%) in lymph node tuberculosis, while the remainder of the cases were negative. In terms of the specimen cell smear test, 11 cases of cancer cells (11/16, 68.8%) were identified in the metastatic tumour case, while there was one case of positive acid-resistant bacilli (1/23, 4.3%) in the lymph node tuberculosis cases. Bacterial cultures were also performed with the control group specimens, with six cases of positive acid-resistant bacilli identified in the lymph node tuberculosis cases (6/19, 31.6%), while the remainder were negative. In terms of cell smear examination, 11 cases of cancer cells (14/18, 77.8%) were identified in the metastatic tumour cases, and two cases were positive for acid-resistant bacilli in the lymph node tuberculosis cases (2/19, 10.5%).

**Table 3:** Comparison of diagnostic effects of puncture cytology and microbiology.

		Bacterial culture *n* (%)	Cell smear *n* (%)
Lymph node tuberculosis	Research group (*n* = 23)	10 (43.5%)	1 (4.3%)
	Control group (*n* = 19)	6 (31.6%)	2 (10.5%)
	*P* value	0.530	0.581
Metastatic tumor	Research group (*n* = 16)	0 (0%)	11 (68.8%)
	Control group (*n* = 18)	0 (0%)	14 (77.8%)
	*P* value	/	0.703

### Comparison of bleeding complications

The only complication across both groups was minimal bleeding, which indicated effective haemostasis following compression. There were no incidences of massive bleeding, postoperative infection or peripheral nerve injury. The bleeding rate was 20.8% (10/48) in the study group and 15.0% (6/40) in the control group, with no significant differences (*P* > 0.05). Thus, the advantage of modified needle aspiration biopsy in terms of few complications was confirmed to be similar to that of CNB (Table 4).

**Table 4:** Comparison of postoperative bleeding rate between two groups of patients.

	Research group (*n* = 48)	Control group (*n* = 40)	*P* value
Bleeding cases	10 (20.8%)	6 (15.0%)	0.480

### Relationship between enlarged diameter of lymph node and diagnostic rate of modified needle aspiration biopsy

With a lymph node diameter of 20 mm set as the boundary, the use of modified needle aspiration biopsy for the diagnosis of lymph node tuberculosis and metastatic tumours was assessed (Table 5). In the diagnosis of lymph node tuberculosis, there was no significant difference in the positive rate of pathological diagnosis and bacterial culture between the two groups (*P* > 0.05), which was also the case for the diagnosis of metastatic tumours in terms of pathological diagnosis and cell smear testing (*P* > 0.05).

**Table 5:** The positive rates of pathological examination, bacterial culture and cytology of modified needle aspiration biopsy in lymph nodes with different diameters.

	Diameter of enlarged lymph nodes	Pathological examination *n* (%)	Bacterial culture *n* (%)	Cell smear *n* (%)
Tuberculosis of lymph nodes	>20 mm (*n* = 17)	14 (82.4%)	9 (52.9%)	1 (5.9%)
	≤20 mm (*n* = 6)	4 (66.6%)	1 (16.7%)	0 (0%)
	*P* value	0.576	0.179	/
Metastatic tumor	>20 mm (*n* = 10)	10 (100%)	0 (0%)	7 (70.0%)
	≤20 mm (*n* = 6)	5 (83.3%)	0 (0%)	4 (66.7%)
	*P* value	0.375	/	0.654

### Clinical evaluation of modified needle aspiration biopsy in the diagnosis of lymphadenectasis disease

Based on the fact that the final diagnosis and the pathological diagnosis of lymphadenectomy presents the gold standard, the sensitivity, specificity, positive predictive value and negative predictive value of the modified needle biopsy method in identifying the benign and malignant conditions of superficial lymphadenopathy were calculated, with the results shown in Table 6. Here, the sensitivity, specificity, positive predictive value and negative predictive values of the modified needle biopsy method were 83.3%, 100%, 100% and 90.9%, respectively.

**Table 6:** Clinical evaluation of modified needle aspiration biopsy in the diagnosis of lymphadenopathy.

		Pathological diagnosis of lymphadenectomy		Total
		Malignant	Benign	
Modified needle aspiration biopsy	Malignant	15	0	15
	Benign	3	30	33
Total		18	30	48
Sensitivity = 15/18*100% = 83.3%				

Sensitivity = 15/18*100% = 83.3%

Specificity = 30/30*100% = 100%

Positive predictive value = 15/15*100% = 100%

Negative predictive value = 30/33*100% = 90.9%

## Discussion

Superficial lymphadenectasis is a common clinical sign, one that can be caused by malignant tumour metastasis, lymph node itself lesions and other diseases. While with the development of imaging technology, the diagnosis and differential diagnosis of lymphadenectasis have been improved, a definitive diagnosis can still not be achieved, and a final pathological diagnosis is needed. As the diagnostic gold standard, lymphadenectomy biopsy involves the issues of long operation time, large trauma, complicated anaesthesia and high cost, making it a controversial procedure for clinical application. The emergence of new technology led to the introduction of the CNB method, which is now widely used in clinical biopsies due to its advantages of slight trauma, few complications and the provision of sufficient tissues for histopathological diagnosis. However, CNB puncture needles are expensive and the puncturing is generally carried out by ultrasound professionals, meaning the method cannot be popularised in clinics and primary medical units. In addition, traditional FNAC has certain limitations for histopathological diagnosis in terms of the fine needle and insufficient tissue.

Most diagnostic reports are based on cytology examinations (6, 10). Therefore, in this study, the negative pressure of needle aspiration biopsy was increased to 20 ml and an 18 G needle was selected as the puncture needle to obtain more tissue specimens, thus meeting the requirements of pathological tissue diagnosis and bacterial culture and cell smear examinations. Furthermore, this method has an advantage over CNB in terms of lower cost due to the use of syringes only, which reduces the cost related to medical consumables. Given that this needle biopsy method was largely inspired by TBNA and was improved based on traditional FNAC, doctors engaged in respiratory intervention can commence quickly, while the method is also suitable for primary medical units.

The TBNA method involves repeated aspiration biopsy using a puncture needle and its safety has been confirmed by numerous clinical data (16). In this study, the puncture needle with the same negative pressure and the same internal diameter as that used in TBNA was used for the aspiration, and the haemostasis was found to be easier when operating on the body surface, meaning the safety of this method is higher than that of TBNA. For certain vascular-rich lymph nodes, it is often difficult to avoid damaging the blood vessels during puncturing. However, during the puncture process, the presence of blood withdrawal in the negative pressure syringe can be observed in real time, and the operation can be stopped at any time.

In this study, all the enrolled patients had no serious complications, such as bleeding or hematoma formation, and there was no statistically significant difference in complication rate between the new method and CNB, indicating that the modified needle aspiration biopsy method has a similar performance as CNB but has better operational safety. Furthermore, this modified needle aspiration biopsy method allowed for obtaining more tissues compared to traditional FNAC, thus meeting the requirements for histopathological diagnosis and consequently improving the diagnostic rate. In this study, the specimens obtained *via* this puncture method had an overall positive rate of 79.2% in histopathological diagnosis, with no significantly statistical difference compared to CNB. In fact, domestic and foreign scholars (17–19) have reported that the overall positive rate of CNB ranges from 79.0% to 95.9%, which is close to the overall positive rate of CNB obtained in the current study. Therefore, we believe that the overall diagnostic positive rate of the modified needle biopsy method is close to that of CNB, which implies that the former has high diagnostic properties.

Hassan et al. (20) reported a CNB positive diagnostic rate of 82.6% in cases with suspected cervical lymph node metastasis of lung cancer. Elsewhere, Oh et al. (21) studied the sensitivity and specificity of CNB in the diagnosis of cervical lymph node metastasis and found them to be 91.6% and 100%, respectively. In the current study, the positive diagnostic rate of CNB for metastatic tumours was 94.4%, while that of the modified needle aspiration biopsy method reached as high as 93.8%, indicating that the latter method has a high positive diagnosis rate in the diagnosis of metastatic tumours in superficial lymph nodes. While the tissue integrity obtained by this method is low, it has less influence on the diagnosis of metastatic tumours, and it is speculated that this modified needle biopsy method is suitable for the initial diagnosis of malignant superficial lymph node metastasis in terms of obtaining sufficient pathological tissue specimens. This specificity for the diagnosis of benign and malignant superficial lymphadenopathy was 100% for this method, similar to that reported for high-specificity CNB. Meanwhile, the sensitivity of the new method was found to 83.3% for the diagnosis of superficial lymphadenopathy, which was lower than that reported for CNB. However, this may have been due to the incorrect diagnosis of lymphoma in the samples. The results of the meta-analysis carried out by Seviar et al. (22) indicated that the effective rate of pathological diagnosis using CNB ranges between 79% and 97% (median 91%) for lymphoma diagnosis, while Oliver et al. (23) concluded that CNB is a reliable method for diagnosing lymphoma through a study of 554 cases. However, Rasmus et al. (24) found that the sensitivity of CNB for lymphoma diagnosis was only 66%, lower than when using surgical resection, and concluded that CNB is not the preferential option for lymphoma diagnosis. In fact, there remains a great deal of controversy surrounding the use of CNB for lymphoma diagnosis.

In the current study, lymphoma was not correctly diagnosed in either group and the following were considered to be possible reasons for this shortfall. First, our unit is an infectious disease specialised hospital, and the pathology department may be relatively weak in terms of the pathological diagnosis of lymphoma. Second, the tissue integrity of the aspiration performed *via* modified needle biopsy is low, potentially affecting the pathological diagnosis of lymphoma. Therefore, modified needle aspiration biopsy is not recommended for the diagnosis of lymphoma.

In China, benign lesions of lymphadenectasis are more common in tuberculosis. Zhao et al. (25) reported that in terms of lymph node tuberculosis, the CNB positive rate when combined with conventional ultrasound guidance was 72.8%, while that of contrast-enhanced ultrasound-guided CNB was 89.7%. In the current study, the pathological diagnostic positive rate of lymph node tuberculosis using modified needle aspiration biopsy was 78.2%, while that when using CNB was 78.9%. Given that the CNB positive rate was close to the reported rate, the diagnosis efficacy of the modified needle aspiration biopsy method is almost equal for lymph node tuberculosis. At the same time, the specimens obtained *via* modified needle biopsy can be submitted for bacteriological cultures in view of conducting further pathogen examinations. The positive rate of the tuberculosis culture of the specimens obtained *via* the puncture biopsy method was only 43.5%. However, while this rate is low, with the development of the Xpert mycobacterium tuberculosis/rifampin assay and metagenomic second-generation sequencing, modified needle aspiration biopsy may have unique advantages over CNB in the diagnosis of lymph node tuberculosis, while the sample size must be expanded in future research.

With regard to the cytology results of the specimens obtained *via* modified needle biopsy, only a high positive rate was obtained in terms of metastatic tumours, while the remaining disease positive rates were low. The use of cytology alone with modified needle biopsy clearly limited the disease diagnosis, and such an approach is not recommended.

Lymph node size may be a potential factor affecting the diagnostic positive rate of needle biopsy. A lymph node diameter of 20 mm was thus adopted as the demarcation to calculate the pathological results, and it was found that there was no significant difference in the positive rate of histopathological diagnosis of lymph node tuberculosis and metastatic tumour, which indicated that the needle biopsy method is less affected by the lymph node size and thus has high applicability.

However, this study also involved a number of limitations. First, this was a single-centre study and the number of collected specimens was small, which may have had some impact on the authenticity of the research conclusions. In addition, only lymph node tuberculosis and metastatic tumours were explored in this study, and no reliable conclusion was achieved regarding the diagnosis efficiency of lymphoma due to the small number of specimens. Therefore, further validation using a multi-centre large-sample study is required.

## Conclusion

Modified needle aspiration biopsy allows for obtaining sufficient histopathological specimens from the enlarged lymph nodes caused by superficial lymph node tuberculosis and metastatic tumours, while the method demonstrates good diagnostic value, potentially providing an effective diagnostic basis for clinical diagnosis. At the same time, the method involves high safety and relatively low costs, with a simple operation, meaning it can be promoted in primary medical units, while the diagnostic value for lymphoma needs to be further explored.

## Data Availability

The original contributions presented in the study are included in the article/Supplementary Material, further inquiries can be directed to the corresponding author/s.
